# Abdominal nonfunctional paraganglioma in which succinate dehydrogenase subunit B (SDHB) immunostaining was performed: a case report

**DOI:** 10.1186/s13256-023-03822-3

**Published:** 2023-03-22

**Authors:** Takazo Tanaka, Akira Joraku, Sayuri Ishibashi, Keisuke Endo, Masahiro Emura, Yusuke Kikuchi, Akito Shikama, Noriko Kimura, Toru Shimazui

**Affiliations:** 1 Department of Urology, Ibaraki Prefectural Central Hospital, 6528, Koibuchi, Kasama, Ibaraki 309-1793 Japan; 2 Department of Endocrinology Diabetes and Metabolism, Ibaraki Prefectural Central Hospital, Kasama, Ibaraki Japan; 3 Department of Pathology, National Hospital Organization Hakodate Hospital, Hakodate, Hokkaido Japan

**Keywords:** Nonfunctioning, Laparoscopic surgery, Genetics, Immunohistochemistry, Paraganglioma

## Abstract

**Background:**

Abdominal nonfunctional paraganglioma is rare. Malignant potential of paraganglioma is assessed by Grading of Adrenal Pheochromocytoma and Paraganglioma score and genetic testing, but genetic testing is not common. We present a case of abdominal nonfunctional paraganglioma whose malignant potential was assessed by grading of adrenal pheochromocytoma and paraganglioma score and succinate dehydrogenase subunit B staining alternative to genetic testing.

**Case presentation:**

A 39-year-old Japanese man had a right retroperitoneal tumor without symptoms. Uptake in the tumor was shown by ^123^I-meta-iodobenzylguanidine scintigraphy. There were no metastases. The results of biochemical workups including blood hormones and urinary metanephrines were normal. We performed retroperitoneoscopic surgery. The tumor was positive for chromogranin A staining but negative for tyrosine hydroxylase. On the basis of the preoperative biochemical workups and pathology results, we diagnosed the tumor as nonfunctional paraganglioma. The Grading of Adrenal Pheochromocytoma and Paraganglioma score classified the tumor as moderately differentiated. Furthermore, negative succinate dehydrogenase subunit B staining suggested the patient has the *SDHx* (*SDHA*, *SDHB*, *SDHC* and *SDHD*) mutation.

**Conclusion:**

Abdominal nonfunctional PGLs are associated with *SDHB* mutation, and SDHB staining should be performed as a screening.

## Background

Paraganglioma (PGL) is a rare disease. The prevalence of pheochromocytoma and PGL in Japan is unknown. Abdominal PGLs originate from sympathetic nerves and therefore secrete catecholamines (functional PGLs) [1, 2]. Rarely, abdominal nonfunctional PGLs occur, but the frequency has not been reported. It was reported that nonfunctional PGLs occurs not only in the paraaortic region, but also in the bladder [3–5].

All PGLs have malignant potential [6]. Malignant potential of PGLs is assessed by the Grading of Adrenal Pheochromocytoma and PGL (GAPP) score, including the initial pathologic and endocrinologic findings [7]. The GAPP score classifies the malignant potential into three levels (well, moderately, and poorly differentiated) and predicts prognosis.

Genetic mutations such as *succinate dehydrogenase subunit B* (*SDHB*), *succinate dehydrogenase subunit D* (*SDHD*), and *rearranged during transfection* (*RET*) have been reported in PGLs [6]. In these genetic mutations, an association between *SDHB* mutation and abdominal nonfunctional PGLs has been reported [1]. On the basis of the first large cohort of Japanese patients with a pheochromocytoma and PGL (PPGL) [8], the proportions of metastasis in PPGL with *SDHB* mutation and without genetic mutation were 36.8% and 13.4%, respectively. Therefore, genetic testing is useful to assess the risk of metastasis, but it is difficult to perform genetic testing in all patients with PPGL. In a previous report, the usefulness of SDHB immunohistochemistry (IHC) staining alternative to genetic testing was reported because defection of SDHB expression indicates *SDHx* (*SDHA*, *SDHB*, *SDHC*, and *SDHD*) mutation [9].

Here, we report a rare case of abdominal nonfunctional PGL. Furthermore, the PGL showed negative SDHB staining, indicating the *SDHx* mutation.

## Case report

**Fig. 1:**
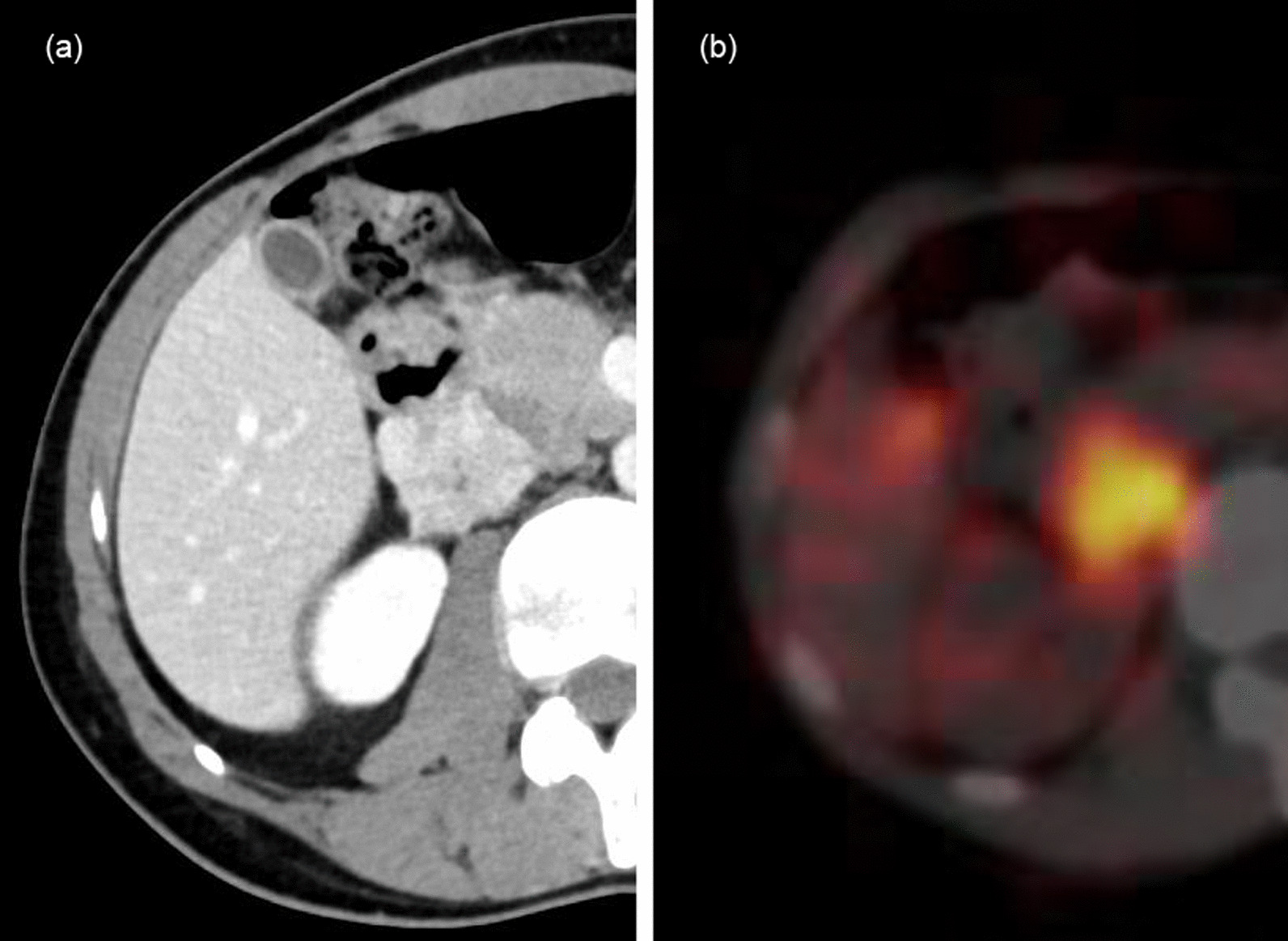
**a** Contrast computed tomography (CT) scan of the abdomen showing a retroperitoneal mass behind the vena cava with an internal hypoabsorption region and contrast enhancement. It bordered on right adrenal glands. **b** ^123^I-MIBG scintigraphy shows accumulation consistent with neoplastic lesions

A Japanese man in his 30s was admitted to our hospital for consultation with a right retroperitoneal tumor detected by ultrasonography in the physical checkup. He had no symptoms, and his physical examination was unremarkable. There was no family history of endocrine disorder or malignancy. Contrast-enhanced computed tomography (CT) scanning revealed a 3.5-cm right retroperitoneal tumor that showed early enhancement post-contrast administration. It was located extra adrenal region and did not originate from the right adrenal gland. Uptake in the tumor was shown through ^123^I-meta-iodobenzylguanidine (MIBG) scintigraphy (Fig. 1a, b). No metastases were found. The 24-hour urine catecholamine, metanephrine, and blood hormone tests all produced normal results. Plasma levels of adrenaline were lower than 0.01 ng/mL (lower than 0.10 ng/mL), noradrenaline was 0.26 ng/mL (0.10–0.50 ng/mL), and dopamine was lower than 0.01 ng/mL (lower than 0.10 ng/mL). Urinary metanephrine was 0.15 mg/day (0.05–0.20 mg/day), and normetanephrine was 0.23 mg/day (0.10–0.28 mg/day).

**Fig. 2:**
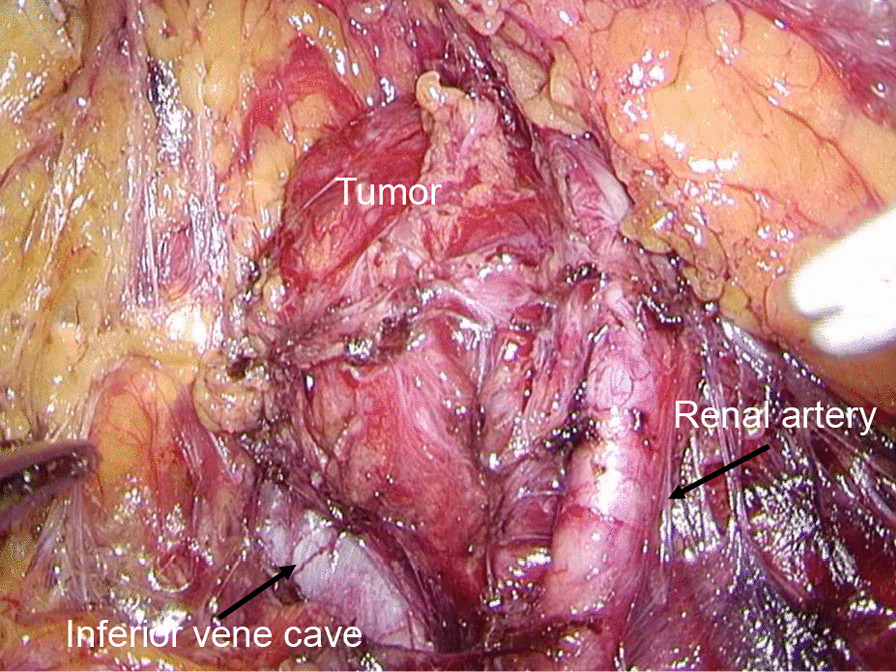
The tumor surrounded by the inferior vena cava and renal artery

Following an initial physical examination, blood and urine tests, and imaging studies, we diagnosed the patient as having a nonfunctional PGL and initiated treatment with an adrenergic alpha 1 receptor antagonist (α1-AR), doxazosin, at 2 weeks prior to surgery. Adverse events such as decreased blood pressure or dizziness did not occur. We then performed a retroperitoneoscopic resection of the tumor. Traffic vessels extended into the tumor from the surrounding inferior vena cava, renal artery, and renal vein (Fig. 2). No enlarged lymph nodes were present. There were no sudden changes in the patient’s blood pressure during the surgery. The tumor was completely resected. The patient was discharged 5 days after surgery.

**Fig. 3:**
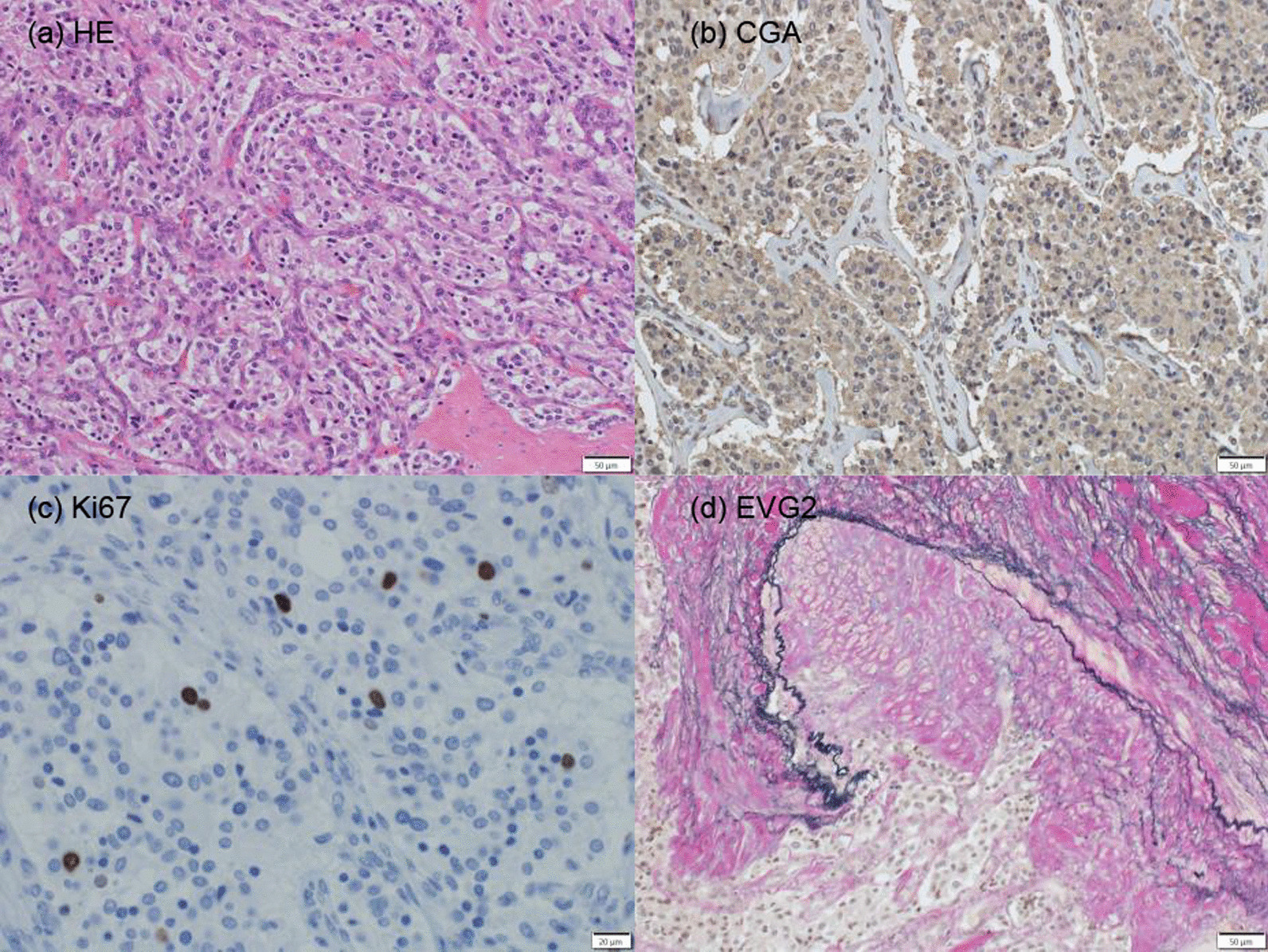
**a** Hematoxylin and eosin (HE) staining demonstrated that the tumor cells were microgranular and contained hyaline globules, and they proliferated in a Zellballen pattern. (HE, ×200) **b** Immunostaining showing high positivity of the tumor cells for chromogranin A (CGA). (CGA, ×200) **c** The presence of Ki67-positive cells in 3% of the tumor. (Ki67, ×400) **d** Elastic van Gieson (EVG) 2-positive cells indicating vascular invasion. (EVG2, ×200)

The tumor’s surface upon splitting was a reddish-brown hue. The Zellballen pattern was shown by hematoxylin and eosin staining of tumor sections. Tumor cells showed positive chromogranin A staining (Fig. 3a, b). The diagnosis of the tumor as a PGL was thus confirmed.

**Fig. 4:**
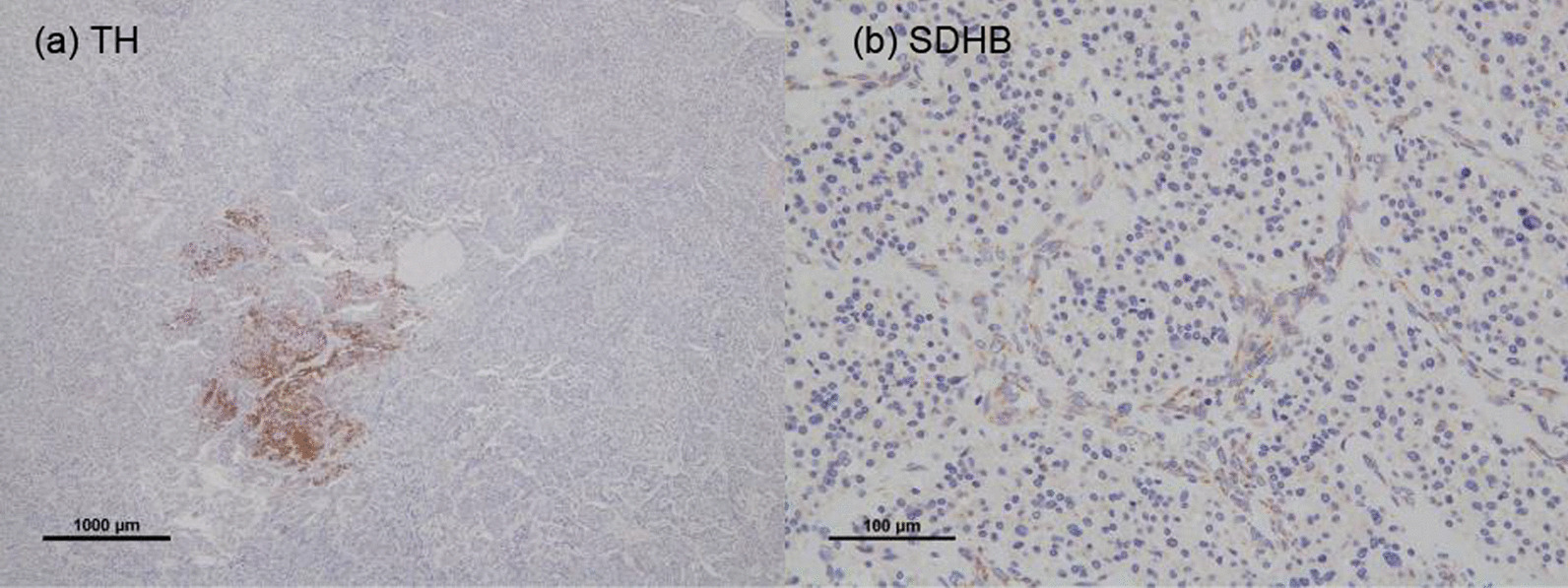
Immunostaining indicating that most of the tumor cells were negative for succinate dehydrogenase subunit B (SDHB) (**a**) and tyrosine hydroxylase (TH) (**b**). (SDHB, ×200; TH, ×20)

It was determined that the GAPP score was 5 due to the cellularity being 300 cells/HPF, a Ki67 labeling index of 3%, and the presence of vascular or capsular invasion (Fig. 3c, d). The tumor was therefore classified as the moderately differentiated type. A further IHC staining of tyrosine hydroxylase (TH) and SDHB was negative, suggesting the tumor was nonfunctional PGL with *SDHx* mutation. (Fig. 4a, b).

After surgery, biochemical workups including blood hormones and urinary metanephrines and CT scans were performed at regular intervals. One year after surgery, no local recurrence or metastasis were observed.

## Discussion

We reported a rare case of abdominal nonfunctional PGL. In prior reports on *SDHx* mutations and catecholamine production, it was established that *SDHB* mutations increase catecholamine production [10], though it was also reported that *SDHB* mutations lead to TH defects in PGL, thus causing nonfunctional PGL [1]. In a report proposing an association between *SDHB* mutation and nonfunctional PGL, 93 out of 182 patients with nonfunctional PPGL and none of the 23 patients with functional PPGL possessed the *SDHB* mutation [11]. In this case, the TH and SDHB staining were negative. These findings and clinical course point to the tumor being a nonfunctional PGL with *SDHx* mutation [9, 12].

Since the WHO classification of endocrine tumors was published in 2017, PPGLs were classified as malignancies [6]. The tumor’s malignant potential was classified by the GAPP score. In this case, GAPP score was 5 and it was moderately differentiated; the reported 5-year survival rate of such tumors is 66.8% (100% for well-differentiated tumors and 22.4% for poorly differentiated tumors) [13]. However, the limitation of GAPP scores that do not include genetic mutations has been pointed out [14]. It has been reported that *SDHB* mutation is associated with malignant PGLs while the malignant potential of PGLs with *RET* mutation is not high [15–18]. Therefore, assessment by GAPP score as well as genetic testing is useful to assess the malignant potential. The efficacy of combining genetic testing and GAPP score was reported [19], however, genetic testing is not commonly performed in clinic due to cost and limited access [9].

The 2022 WHO classification recommend SDHB staining for PPGL because defection of SDHB expression indicates *SDHx* mutation [6]. The sensitivity and specificity of SDHB staining are 100% and 84%, respectively [9]. Some reports have attempted to use a combination of SDHB staining and GAPP score as a better indicator of malignant potential [7, 20]. We performed SDHB staining as a screening of genetic mutation because it was difficult to conduct genetic testing, and we could obtain more information of the malignant potential and estimate the risk of onset in the family.

## Conclusions

We showed a case of abdominal nonfunctional PGL. *SDHB* mutation causes nonfunctional PGL and high malignant potential of tumor. Performing SDHB staining is simple and can be an alternative to genetic testing for PGL diagnosis.

## Data Availability

Data sharing is not applicable to this article as no new data were created or analyzed in this study.

## Abbreviations

CT: Computed tomography
EVG2: Elastica van Gieson
GAPP: Grading of Adrenal Pheochromocytoma and Paraganglioma
HPPS: Hereditary pheochromocytoma-paraganglioma syndrome
MIBG: Meta-iodobenzylguanidine
PGL: Paraganglioma
PPGL: Pheochromocytoma and paraganglioma
RET: Rearranged during transfection
SDHB: Succinate dehydrogenase subunit B
TH: Tyrosine hydroxylase
α1-AR: Adrenergic alpha 1 receptor antagonist
IHC: Immunohistochemistry
